# Using Selection by Nonantibiotic Stressors to Sensitize Bacteria to Antibiotics

**DOI:** 10.1093/molbev/msz303

**Published:** 2019-12-18

**Authors:** Jeff Maltas, Brian Krasnick, Kevin B Wood

**Affiliations:** 1 Department of Biophysics, University of Michigan, Ann Arbor, MI; 2 Department of Physics, University of Michigan, Ann Arbor, MI

**Keywords:** microbial evolution, antibiotic resistance, collateral sensitivity

## Abstract

Evolutionary adaptation of bacteria to nonantibiotic selective forces, such as osmotic stress, has been previously associated with increased antibiotic resistance, but much less is known about potentially sensitizing effects of nonantibiotic stressors. In this study, we use laboratory evolution to investigate adaptation of *Enterococcus faecalis*, an opportunistic bacterial pathogen, to a broad collection of environmental agents, ranging from antibiotics and biocides to extreme pH and osmotic stress. We find that nonantibiotic selection frequently leads to increased sensitivity to other conditions, including multiple antibiotics. Using population sequencing and whole-genome sequencing of single isolates from the evolved populations, we identify multiple mutations in genes previously linked with resistance to the selecting conditions, including genes corresponding to known drug targets or multidrug efflux systems previously tied to collateral sensitivity. Finally, we hypothesized based on the measured sensitivity profiles that sequential rounds of antibiotic and nonantibiotic selection may lead to hypersensitive populations by harnessing the orthogonal collateral effects of particular pairs of selective forces. To test this hypothesis, we show experimentally that populations evolved to a sequence of linezolid (an oxazolidinone antibiotic) and sodium benzoate (a common preservative) exhibit increased sensitivity to more stressors than adaptation to either condition alone. The results demonstrate how sequential adaptation to drug and nondrug environments can be used to sensitize bacteria to antibiotics and highlight new potential strategies for exploiting shared constraints governing adaptation to diverse environmental challenges.

## Introduction

The emergence of drug resistance is continually shrinking an ever-smaller pool of drugs necessary for the successful treatment of infectious disease (Levy and Marshall 2004; Boucher et al. 2009; Borst 2012; Goldberg et al. 2012; Pfaller 2012; Pluchino et al. 2012; Raviglione et al. 2012). The evolution of resistance is a complex stochastic process that may depend on spatiotemporal dynamics of the host environment (Zhang et al. 2011; Greulich et al. 2012; Hermsen et al. 2012; Fu et al. 2015; Moreno-Gamez et al. 2015; Baym, Lieberman, et al. 2016; De Jong and Wood 2018). In addition, resistance evolution in fluctuating or multiagent environments may be driven by phenotypic trade-offs reflecting conflicting evolutionary goals. For example, recent studies have shown that acquiring resistance to a single antibiotic frequently leads to a change in the susceptibility to a different antibiotic, a phenomenon known as collateral sensitivity or cross-resistance (Imamovic and Sommer 2013; Kim et al. 2014; Lazar et al. 2013, 2014; Munck et al. 2014; Oz et al. 2014; de Evgrafov et al. 2015; Barbosa et al. 2017; Dhawan et al. 2017; Yoshida et al. 2017; Maltas and Wood 2019; Nichol et al. 2019; Rosenkilde et al. 2019). Although the majority of the focus has been on in vitro evolution experiments, recent studies have also demonstrated collateral effects in clinical in vivo systems (Jansen et al. 2016; Podnecky et al. 2018). Although the molecular mechanisms of collateral sensitivity have been identified in several specific cases—for example, modulation of proton-motive force underlies increased sensitivity to some antibiotics induced in aminoglycoside-resistant mutants (Lazar et al. 2013)—they are generally difficult to uncover and may vary by species and drug, making them an ongoing focus of research. At the same time, a number of recent studies have shown that system-level approaches based on phenotypic profiling may help identify statistical properties of these collateral effects, even when molecular mechanisms are not fully known (Imamovic and Sommer 2013; Lazar et al. 2014; Oz et al. 2014; Dhawan et al. 2017; Barbosa et al. 2018; Yoon et al. 2018; Maltas and Wood 2019). Additionally, recent work reveals the temporal stability of collateral sensitivity and highlights the potential of collateral sensitivity-based treatment strategies (Barbosa et al. 2019).

In addition to antibiotics, many studies have shown that exposure to nonantibiotic conditions, such as heavy metals, biocides, extreme temperatures, acidic or osmotic stress, and even growth media may also lead to reduced susceptibility to antimicrobials (Hassen et al. 1998; Baker-Austin et al. 2006; Yazdankhah et al. 2006; Horner et al. 2012; Ji et al. 2012; Seiler and Berendonk 2012; Carey and McNamara 2015; Knöppel et al. 2017; Wand et al. 2017; Bhardwaj et al. 2018). For example, adaptation to the antiseptic chlorhexidine (CHX) was recently shown to be associated with collateral resistance to daptomycin, a lipopeptide antibiotic used to treat multidrug-resistant Gram-positive infections (Bhardwaj et al. 2018). On the other hand, antibiotic-resistant strains may exhibit increased sensitivity to antimicrobial peptides (Lázár et al. 2018), and bacteria undergoing long-term evolution without drug generally show decreased antibiotic resistance (Lamrabet et al. 2019). As a whole, these studies point to overlapping evolutionary constraints that govern adaptation to a large and chemically diverse collection of deleterious environments. In turn, they raise the question of whether nonantibiotic stressors—which are frequently encountered in both clinical and natural environments—might play an important role in the evolution of drug resistance and, at the same time, represent an untapped set of environmental “levers” for steering evolutionary trajectories (Nichol et al. 2015).

Although there has been extensive progress identifying the molecular mechanisms governing cross-resistance between specific pairs of antibiotic and nonantibiotic stressors, relatively little is known about the system-level properties of these evolutionary trade-offs. Phenotypic studies may complement mechanistic approaches by identifying statistical relationships between large collections of stressors, offering new insights into questions that are difficult to answer from molecular information alone. For example, does adaptation to nonantibiotic stressors frequently lead to modulated antibiotic resistance, or are these effects relatively rare, restricted—perhaps—to structurally or mechanistically similar agents? When these collateral effects appear, are they dominated by cross-resistance, pointing to an ever-accelerating march to resistant pathogens with broad multiagent resistance? Or do these conditions co-select for increased sensitivities, potentially leading to multiagent environmental sequences that trap cells in evolutionarily vulnerable states? Recent evolution-based approaches have revolutionized our view of multidrug therapies (Baym, Stone, et al. 2016). Nonantibiotic stressors may offer a complementary set of unappreciated selective forces for simultaneously sensitizing pathogens to multiple drugs.

In this work, we start to answer some of these questions using laboratory evolution and phenotypic profiling in an opportunistic bacteria pathogen. Specifically, we investigate phenotypic collateral effects arising during bacterial adaptation to six antibiotics and seven nonantibiotic environments, including common biocides, extreme pH, and osmotic stress. As a model system, we focus on *Enterococcus* *faecalis*, a Gram-positive bacterial species frequently found in the gastrointestinal tracts of humans. *Enterococcus* *faecalis* can survive in a range of harsh environments, making it a good candidate for adaptation to many different environmental conditions. At the same time, *E. faecalis* is an important clinical pathogen that contributes to multiple human infections, including urinary tract infections and infective endocarditis (Cetinkaya et al. 2000; Donlan 2001; Clewell et al. 2014; O’Driscoll and Crank 2015).

In a recent study, we used laboratory evolution to characterize the phenotypic collateral sensitivity profiles between multiple antibiotics in *E. faecalis* (Maltas and Wood 2019). In this study, we show that collateral resistance and sensitivity are also surprisingly common between more general environmental stressors, both between different nonantibiotic stressors and between antibiotics and nonantibiotic conditions. Although the specific resistance profiles vary between independent populations, even when selected by the same condition, the collateral sensitivities remain common. For example, 25 of 32 isolates selected by the antimicrobial triclosan (TCS) exhibited increased sensitivity to at least one of the six antibiotics tested. Finally, we show experimentally that populations evolved to a sequence of two conditions (the antibiotic linezolid [LZD] and the preservative sodium benzoate [NaBz]) can induce increased sensitivity to more conditions than adaptation to either stressor alone. The results demonstrate how sequential adaptation to drug and nondrug environments can be used to sensitize bacteria to antibiotics and highlight new potential approaches for leveraging evolutionary trade-offs inherent in adaptation to diverse environments.

## Results

### Collateral Effects between Antibiotic and Nonantibiotic Stressors Are Common

**Fig. 1.**
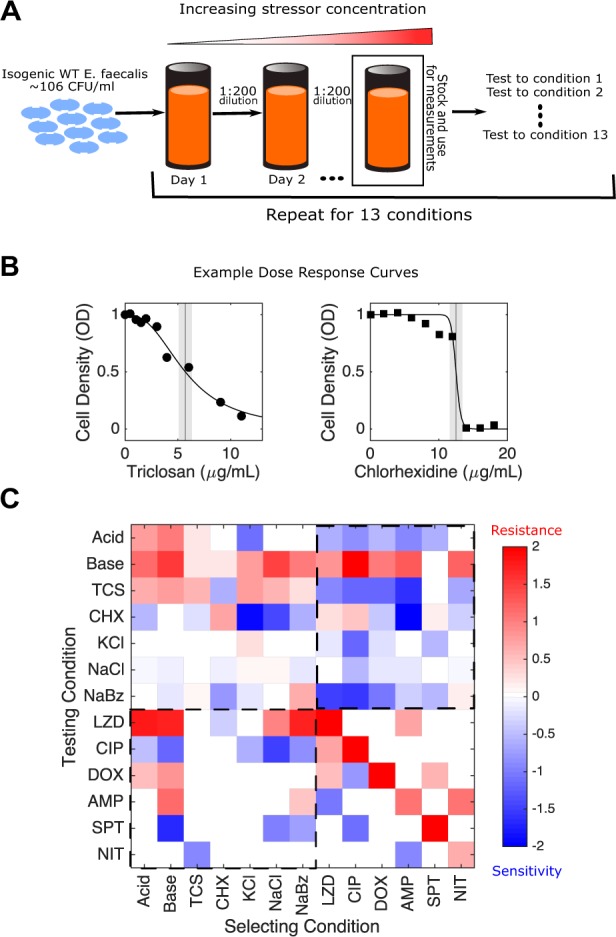
Laboratory evolution reveals collateral sensitivity and cross-resistance between antibiotics and environmental stressors in *Enterococcus faecalis*. (*A*) Populations of *E. faecalis* V583 were exposed to increasing concentrations of a single selecting condition over multiple days via serial passage experiments (Materials and Methods). The evolution was repeated for 13 different selecting conditions, including six antibiotics and seven nonantibiotic stressors (table 1). At the end of the evolution experiment, a single colony was isolated from each population and tested for modulated sensitivity to each of the 13 environmental conditions. (*B*) Example dose–response curves for isolates selected by TCS (left) and chlorhexidine (right). Vertical line represents estimated half-maximal inhibitory concentration (IC_50_), with shaded regions confidence intervals (95%). (*C*) Resistance (red) or sensitivity (blue) to each condition is quantified using the (log_2_-transformed) fold change in the IC_50_ for the selected isolate relative to that of ancestral (V583) cells. Dashed regions correspond to antibiotic susceptibilities of nonantibiotic selected isolates (lower left) and, conversely, nonantibiotic susceptibilities of antibiotic selected isolates (upper right).

To investigate collateral effects between antibiotic and nonantibiotic conditions, we exposed populations of *E. faecalis* strain V583, a clinical isolate (Paulsen et al. 2003), to increasing concentrations of a single condition for up to 60 days (∼450 generations) via serial passage evolution (fig. 1*A*; Materials and Methods). We repeated this laboratory evolution for 13 different selecting conditions, including extreme pH, osmotic stress, biocides, preservatives, and traditionally known antibiotics (table 2). Following laboratory evolution, we isolated a single colony (“mutant”) from each population and measured its susceptibility to all seven conditions as well as to six antibiotics spanning multiple classes via high-throughput dose–response experiments. In addition, we measured susceptibility of six previously isolated strains (one for each antibiotic; strains were originally isolated in Maltas and Wood [2019]) to all seven nonantibiotic stressors. To quantify resistance to each condition, we estimated the half maximal inhibitory concentration (IC_50_) for all 13 isolates, as well as isolates from the ancestral populations, to each of the 13 conditions (Materials and Methods; fig. 1*B*). In total, we estimated the IC_50_ for 170 isolate-condition combinations (each performed in technical replicates of three). For each isolate-condition combination, we then calculate $c\equiv {\;\text{log}\;}_{2}\left({\text{IC}}_{50,\text{Mut}}/{\text{IC}}_{50,\text{WT}}\right)$, the log-scaled fold change in IC_50_ of the mutant (relative to ancestral strains) (fig. 1*C*). Resistance therefore corresponds to *c *>* *0 and sensitivity to *c *<* *0. To minimize false positives, only *c* values larger than three *σ*_WT_ were deemed to have collateral sensitivity or collateral resistance, where *σ*_WT_ corresponds to the standard error of the mean across three technical replicates of the wild type.

**Table 2.** Selecting Conditions Used in This Study.

Condition	Class	Mechanism of Action
Ampicillin (AMP)	*β*-Lactam	Inhibits cell-wall synthesis
Doxycycline (DOX)	Tetracycline	30S protein synthesis inhibitor
Spectinomycin (SPT)	Aminoglycosides	30S protein synthesis inhibitor
Linezolid (LZD)	Oxazolidinone	50S protein synthesis inhibitor
Ciprofloxacin (CIP)	Quinolone	DNA gyrase inhibitor
Nitrofurantoin (NIT)	Nitrofuran	Multiple mechanisms
Chlorhexidine (CHX)	Biocide	Disrupts the cell membrane
Triclosan (TRC)	Biocide	Inhibits fatty acid synthesis
Sodium Benzoate (NaBz)	Preservative	Decreases intracellular pH
Alkaline pH (Base)	N/A	N/A
Acidic pH (Acid)	N/A	N/A
Potassium chloride (KCl)	N/A	N/A
Sodium chloride (NaCl)	N/A	N/A

Note.—N/A, not applicable.

We find that isolates selected by antibiotics frequently exhibit modulated sensitivity to nonantibiotic conditions, and conversely, isolates selected by nonantibiotics often exhibit modulated sensitivity to antibiotics (fig. 1*C*). Sensitivity was altered in 62% (104/169) of condition-mutant pairs, with 58% (91/156) corresponding to collateral effects (i.e., modulated resistance to a stressor other than that used for selection). Collateral sensitivity is more common (58%, 53/91) than collateral resistance (42%, 38/91), though all 13 isolates exhibited both collateral resistance and collateral sensitivity to at least two distinct conditions. A histogram of all measured *c* resistance values (supplementary fig. S4*A*, Supplementary Material online), as well as the fraction of collaterally sensitive and resistant observations over a wide range of different cutoff values, is available in the Supplementary files (supplementary fig. S4*B*, Supplementary Material online). NaCl and KCl appear to have only acquired modest resistance. It is possible that resistance acquisition may be slower to accumulate as *Enterococcus* is naturally tolerant to many harsh environments, including osmotic stress (Gilmore et al. 2002; Solheim et al. 2014).

**Fig. 2.**
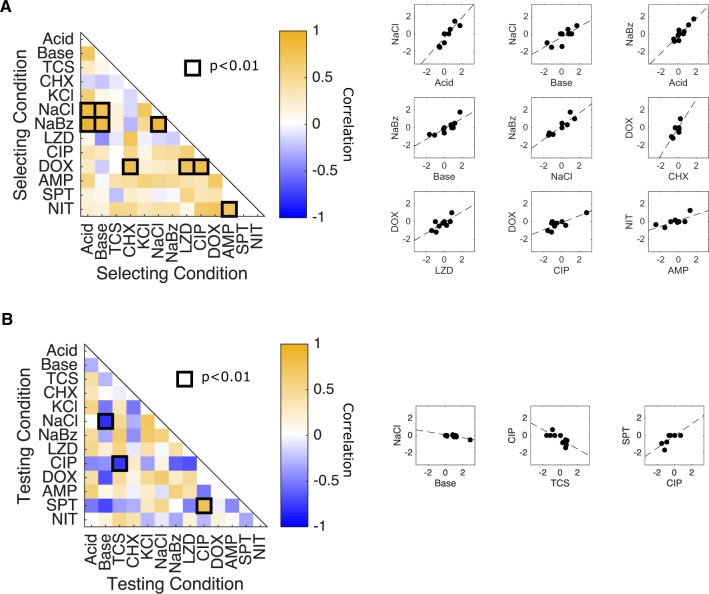
Correlations between collateral effects under different selecting or testing conditions. (*A*) Left panel: Pearson correlation coefficient between collateral profiles (i.e., columns of the matrix in fig. 1*C*) selected under different conditions. Dark squares highlight significant correlations (*P *<* *0.01), which are also shown as scatter plots. Right panels: pairwise scatter plots of resistance profiles selected by different conditions (i.e., scatter plots comparing columns of the matrix in fig. 1*C*; only pairs with significant correlations, *P *<* *0.01, are shown). Each point represents the measured resistance to a single stressor in isolates selected by the conditions on the horizontal and vertical axes, that is, each point is the resistance value *c* in each of the two conditions labeled by the axis. (*B*) Left panel: Pearson correlation coefficient between resistance levels to a particular testing condition (i.e., rows of the matrix in fig. 1*C*) across the ensemble of isolates. Dark squares highlight significant correlations (*P *<* *0.01). Right panels: pairwise scatter plots of resistance levels to different conditions (i.e., scatter plots comparing rows of the matrix in fig. 1*C*; only pairs with significant correlations, *P *<* *0.01, are shown). Each point represents a single isolate and shows the resistance of that isolate to the pairs of testing conditions on the horizontal and vertical axes. Put another way, each point represents the value *c* to each condition on the axis. To remove the effects of direct selection, which are typically larger in magnitude and may bias the correlations, the diagonal entries of the collateral sensitivity matrix (corresponding to resistance to the selecting condition) are removed prior to calculating all correlations. In all cases, resistance is measured in units of (log_2_-scaled) fold change in IC_50_ relative to ancestral strain (see supplementary figs. S1 and S2, Supplementary Material online, for scatter plots for all pairs).

We next asked whether the resistance profiles selected by different conditions show statistical similarities. Here, a resistance profile of a selecting condition is defined by a column in figure 1*C*. One might hypothesize, for example, that profiles selected by chemically similar stressors would be strongly correlated with one another. On the other hand, correlations between profiles could also arise if different stressors are associated with molecularly promiscuous resistance determinants—for example, multidrug efflux pumps (Lee et al. 2003) that extrude unrelated chemical stressors. Indeed, we found strong correlations between the resistance profiles selected under many different pairs of conditions (fig. 2*A*). For example, profiles selected by NaCl are significantly correlated with those selected by acidic conditions, basic conditions, and NaBz. In addition, profiles selected by doxycycline (DOX), a protein synthesis inhibitor, are correlated with those selected by other structurally dissimilar compounds, including two antibiotics (LZD and ciprofloxacin [CIP]) as well as the antiseptic CHX. Overall, correlations between pairs of selecting conditions are dominated by positive correlations (62/78 pairs), including in all nine pairs eclipsing the significance (*P *<* *0.01) threshold. Similarly, we asked whether resistance levels between pairs of different testing conditions were correlated across different isolates. Here, a resistance profile of a testing condition is defined as a row in figure 1*C*. We found positive correlations to also be slightly more common between testing conditions (45/78 pairs), though two of the three pairs eclipsing significance (*P *<* *0.01) exhibited negative correlations. Specifically, we found negative correlations between resistance to NaCl and basic conditions and between CIP and TCS, but positive correlations between CIP and spectinomycin (SPT) (see also supplementary figs. S1 and S2, Supplementary Material online, for scatter plots between all pairs of selecting and testing conditions, respectively).

### Strains Selected by Nonantibiotic Pressures Often Carry Mutations in Genes Known to Confer Antibiotic Resistance or Sensitivity

To identify candidate genes that may underlie changes in sensitivity to one or more environments, we performed whole-genome sequencing on both single isolates (a single colony selected from an agar plate) and population samples (well-mixed 200 *μ*l samples) from the evolved populations. Specifically, we sequenced single isolates from each evolved population, an isolate evolved in media (Brain heart infusion [BHI]) only for 8 days, and also two individual isolates from the ancestral strains. In addition, we performed population sequencing on a sample from each population, including the media-selected control. Because the number of variant calls rises rapidly for small mutation frequencies (supplementary fig. S3, Supplementary Material online), we limit our analysis to variants estimated to occur with frequency >30% (Materials and Methods). Note that samples from four of the antibiotic-selected populations (those selected by ampicillin [AMP], DOX, nitrofurantoin [NIT], and SPT) and the BHI-population were sequenced for a previous study (Maltas and Wood 2019) and their results are included here for comparison. In addition, we exclude sequencing from the acid-selected population, which was contaminated during preparation for sequencing, and exclude variants occurring in all sequenced strains. As a final control, we also confirmed a small number of mutations (in *rpsJ* in the DOX-selected isolate and in *parC* in the CIP-selected isolate) via polymerase chain reaction amplification and Sanger sequencing.

Overall, we observe significant agreement between population and single isolate sequencing; every mutation that occurs in the clonal sample also occurs with at least 68% frequency in the corresponding population sample (table 1). Similarly, all mutations occurring at a frequency of at least 90% in the population samples also occur in the clonal sequences. Despite this agreement, we do observe apparent heterogeneity in several populations (e.g., LZD).

**Table 1.** Mutations Identified in Selected Populations.

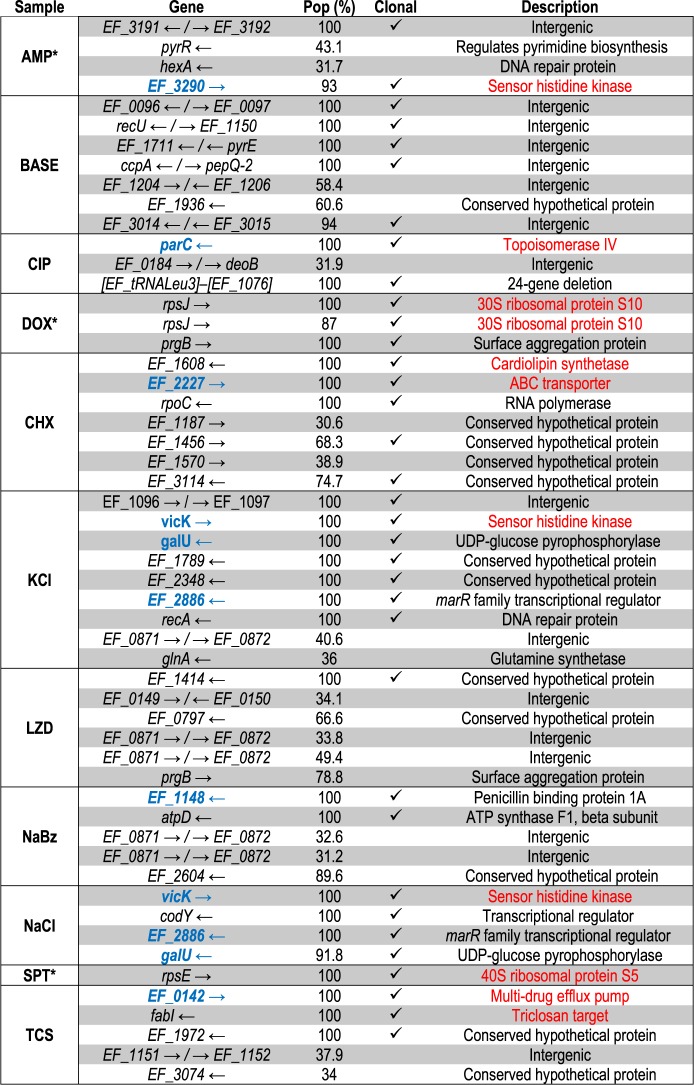

Note.—Mutations listed in red have been previously linked with resistance to the selecting condition, whereas genes listed in blue are genes previously identified with resistance to environments other than the selecting condition.

a Strains evolved and sequenced for previous work.

This consistency suggests that the phenotypic measurements, performed on single clones isolated from each population, are generally representative of the entire population, though more substantial population heterogeneity is apparent in a several cases (e.g., LZD).

This analysis reveals 54 mutations achieving at least 30% frequency in at least one population. All mutations identified were nonsynonymous. Using this 30% threshold, we see as many as nine mutations in a strain (KCl) and as few as zero (NIT). Although we observe no mutations in the NIT strain, repeated experiments confirm increased resistance. Because the resistance is relatively small (less than a factor-2 increase), it is possible that the observed resistance stems from transient phenotypic resistance related to the postantibiotic effect or cellular hysteresis observed when rapidly cycling drugs (Roemhild et al. 2018). The control population selected by media alone showed no mutations above 30% frequency. Fifty-two of the 54 mutations occurred on the chromosome, whereas both *prgB* mutations occurred on the pTEF2 plasmid. In addition, we see several mutations present in genes known to confer resistance to the selecting drug. For example, the CHX-selected population contains two previously identified mutations responsible for CHX resistance, one in *EF_1608*, a cardiolipin synthetase, and one in *EF_2227*, an ATP-binding cassette transporter (Bhardwaj et al. 2018; Li and Palmer 2018). The TCS isolate contains a mutation in *fabI*, a common TCS-resistant gene and the drug’s target (Heath et al. 1999), as well as *EF_0142*, an efflux multidrug-resistant transporter (Kumar et al. 2013). We identify a shared mutation between KCl and NaCl in *vicK*, a sensor histidine kinase known to confer resistance to environments such as osmotic stress, pH, and temperature (Senadheera et al. 2009). In addition, the DOX-selected population contains two mutations in *rpsJ*, a gene known to confer resistance to the tetracycline class of antibiotics (Beabout et al. 2015), and the CIP-selected population contains a *parC* mutation, a gene known to confer high levels of CIP resistance (Belland et al. 1994), both of which were reported previously (Maltas and Wood 2019).

In addition to mutations present in genes linked with resistance to the selecting condition, we also identified mutations in genes potentially responsible for modulated sensitivity to nonselecting conditions, including mutations to genes known to confer antibiotic resistance in populations selected by nonantibiotic stressors. For example, the NaCl- and KCl-selected populations harbor a mutation in *EF_2886*, an *marR* family transcriptional regulator. The *marR* system is known to regulate efflux pump activity and underlies resistance to a wide range of structurally diverse drugs (Levy 1992; Hao et al. 2014; Sharma et al. 2017). Recent work indicates that increased efflux activity comes with a trade-off, as corresponding changes in the proton-motive force can induce sensitivity to aminoglycoside antibiotics (Lazar et al. 2014). Consistent with these findings, we find that an isolate selected by NaCl exhibited increased sensitivity to the aminoglycoside SPT. The *marR* system is also known to confer resistance to oxidative stress, similar to TCS (Hao et al. 2014; Lu et al. 2018); it is perhaps not surprising, then, that we observe TCS resistance in populations selected by either NaCl or KCl. We also identify a mutation in *EF_1148*, a penicillin-binding protein, in isolates selected by NaBz. Mutations in penicillin-binding proteins are known to confer resistance to *β*-lactam antibiotics (Arbeloa et al. 2004), and indeed we observe cross-resistance to AMP in isolates from the NaBz-selected population.

Finally, we identify mutations in genes that have been previously linked with collateral sensitivity or resistance to antibiotics, though we observe phenotypes that differ from those expected. For example, the *marR* mutation in KCl and NaCl, the *EF_2227* mutation in CHX, and the *EF_0142* mutation in TCS are all related to efflux pumps, which are known to confer resistance to a wide array of antibiotics and biocides, especially tetracyclines and quinolones (Lazar et al. 2014; Sharma et al. 2017); surprisingly, we see no increased resistance to these antibiotics in the corresponding isolates (fig. 1). Additionally, NaCl and KCl share a mutation in *galU* which is known to confer pleiotropic effects (Rigottier-Gois et al. 2011) and AMP resistance (Eriksson-Grennberg et al. 1971), though we see no increase in AMP resistance in isolates from the same population (fig. 1). These discrepancies could arise for several reasons. First, although we observe mutations in genes linked with drug resistance, the specific mutations are not necessarily the same. For example, the study of *EF_2227* focuses on the full gene knockout while we observe a single nonsynonymous substitution (Li and Palmer 2018). On the other hand, the discrepancies could also be explained by epistatic effects that potentially differ in different genetic backgrounds, giving rise to variable phenotypes (Whitlock et al. 1995; Trindade et al. 2009; Schenk et al. 2013; Weinreich et al. 2013; Ogbunugafor et al. 2016; Sommer et al. 2017; Card et al. 2019; Guerrero et al. 2019). It is possible that the isolate selected for phenotyping represents a rare variant of the population and therefore is not well described by the population sequencing, though the relatively high frequencies estimated for most variants suggest that this explanation is unlikely in many cases. A full list of all identified mutations is available with more details in the Supplementary Material online.

### Selection by CHX or TCS Frequently Sensitize Bacteria to At Least One Antibiotic

**Fig. 3.**
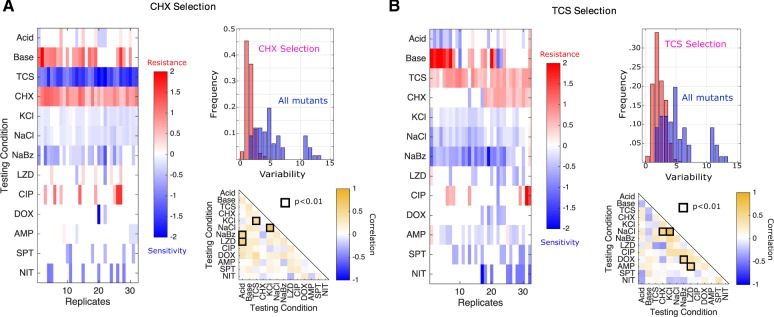
Isolates selected by CHX or TCS are variable but often exhibit increased sensitivity to antibiotics. Collateral resistance profiles for 32 independent populations evolved to either CHX (*A*) or TCS (*B*). Left panels: resistance (red) or sensitivity (blue) to each condition (rows) is quantified using the (log_2_-transformed) fold change in the IC_50_ for the selected isolate relative to that of ancestral (V583) cells. Color scale ranges from −2 (4× decrease in IC_50_, blue) to +2 (4× increase in IC_50_, red). Top right: histogram of variability in collateral profiles for isolates selected by CHX/TCS (red) or for the isolates in figure 1 (spanning all conditions, blue). Variability for each collateral profile is defined as the Euclidian distance between that profile and the centroid formed by the relevant ensemble of profiles. Mean variability differs between isolates selected by CHX and all isolated mutants, as well as between isolates selected by TRC and all isolated mutants, each with *P *<* *0.001 (*t*-test with unequal variance). Bottom right: Pearson correlation coefficient between resistance levels to a particular testing condition across the ensemble of isolates.

Previous studies have shown that collateral profiles may be highly variable, even when selection is performed multiple times under the same conditions (Maltas and Wood 2019; Nichol et al. 2019). To estimate this variability for nonantibiotic stressors, we evolved 32 replicate populations to each of two antimicrobials, TCS and CHX, for a total of 22 days (∼170 generations). TCS is an antimicrobial agent found in numerous consumer products, including soaps, body washes, and toothpastes. It has been linked with cross-resistance to antibiotics in multiple species (Yazdankhah et al. 2006) and was recently shown to induce resistance to antibiotics both in vitro and in vivo (Westfall et al. 2019). CHX is an antimicrobial found in many disinfectants and commonly used as a general antiseptic in hospitals. CHX exposure has been linked with increased resistance to daptomycin in *E. faecium*, a closely related enterococcal species (Bhardwaj et al. 2018). Following the laboratory evolution to each condition, we measured the resistance profiles for single isolates from each population to all 13 environmental conditions (fig. 3). Surprisingly, isolates selected by each condition frequently exhibit collateral sensitivity to other agents, with 15/32 CHX isolates and 25/32 TCS isolates showing sensitivity to at least one antibiotic. In addition, all 32 CHX isolates showed strong sensitivity to TCS, whereas half of the 32 TCS isolates show cross-resistance to CHX.

To quantify variation within an ensemble of collateral profiles, we considered each profile as a 13D vector, with each component representing resistance to a particular environmental condition. To estimate variability within the ensemble, we calculated the mean pairwise (Euclidean) distance, $\langle d_{p}\rangle$, across all pairs of profiles in the ensemble. Although collateral profiles of isolates selected by TCS ($\langle d_{p}\rangle =2.2$) and CHX ($\langle d_{p}\rangle =1.6$) both exhibit isolate-to-isolate variability, it is considerably smaller than the variability observed across all conditions ($\langle d_{p}\rangle =5.2$). In addition, the distribution of pairwise distances between isolates selected by the same condition (TCS or CHX) is considerably narrower than the distribution across all isolates (fig. 3, upper right insets). We also tested for correlations between resistance levels to pairs of stressors across the ensemble of isolates for each condition. Not surprisingly, the correlations between pairs of stressors vary substantially depending on the selecting conditions used to generate the isolates (compare insets in fig. 3*A* and *B*). For example, resistance to KCl is correlated with resistance to TCS following CHX selection (fig. 3*A*, lower right) but weakly anticorrelated in TCS-selected isolates (fig. 3*B*, lower right). On the other hand, there are rare pairs of environments—such as NaCl and KCl—where resistance is strongly correlated in all sets of isolates, likely reflecting the extreme chemical similarity between the stressors.

### Sequential Rounds of Antibiotic and Nonantibiotic Selection Can Promote Sensitivity

Our results indicate that both collateral sensitivity and cross-resistance are surprisingly common in the evolved lineages. Selection by one condition (by definition) leads to resistance to that condition, but it frequently sensitizes the population to multiple other conditions. In fact, our experiments showed that selection by one stressor led to increased sensitivity to between three and seven other conditions (fig. 1*B*). Unfortunately, these increased sensitivities are also accompanied by frequent cross-resistance, placing limits on the number of sensitivities that can be selected by any one condition.

**Fig. 4.**
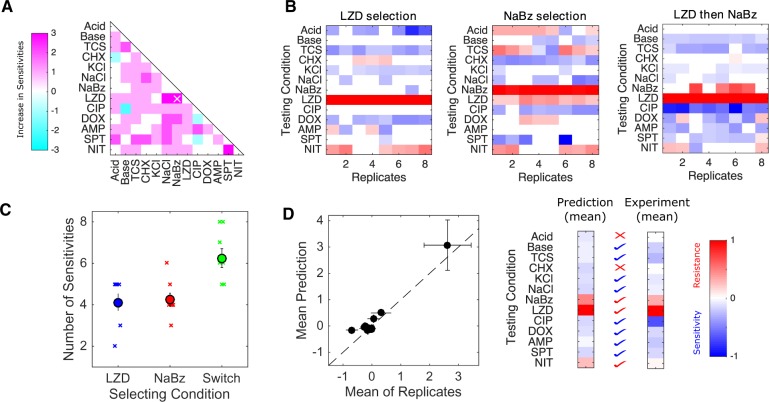
Selection in alternating environments can induce sensitivity to more stressors than selection in single environments. (*A*) Predicted change in number of sensitivities following sequential evolution using pairs of conditions. Change is positive if sequential evolution is predicted to result in more sensitivities than evolution in either component alone. Predictions assume additivity of (log-scaled) resistance profiles. A sequence of LZD and NaBz (white x) is predicted to give the maximum increase in sensitivities. (*B*) Resistance profiles for replicate evolution experiments (eight per condition) in LZD only, NaBz only, or a two-step sequence consisting of LZD selection followed by NaBz selection. Resistance (red) or sensitivity (blue) to each condition (rows) is quantified using the (log_2_-transformed) fold change in the half-maximal inhibitory concentration (IC_50_) for the selected isolate relative to that of ancestral (V583) cells. (*C*) Left panel: isolates evolved in the alternating environment (“switch” between LZD then NaBz, green) exhibit sensitivity to more environments than isolates selected in each environment alone (blue and red; *P *<* *0.01, Wilcoxon rank sum test for pairwise comparisons between LZD and switch and between NaBz and switch. (*D*) Left: scatter plot comparing the mean collateral profile of isolates from the alternating selection (“experiment”) and mean collateral profiles predicted by an average (linear sum) of profiles generated in the single environments (“prediction”). Right panel: heat maps (color scale same as for panel *A*). Check marks indicate correctly predicted sensitivity (blue) or resistance (red). X indicates incorrect prediction.

However, we hypothesized that it might be possible to circumvent those limitations by using a sequence of two stressors. Although this sequential selection is likely to produce resistance to, at minimum, the two selecting conditions, it is possible that judiciously chosen conditions could lead to more sensitivities than either condition alone—in effect harnessing the orthogonal sensitizing effects of particular pairs of selective forces. To guide our search, we first calculated the expected number of sensitivities following sequential selection by each pair of conditions under the naive assumption that phenotypic effects are purely additive. Because resistance is measured on a log scale, the assumption of additivity means that relative changes in IC_50_ (or similar) are multiplicative; for example, if conditions 1 and 2 each reduce IC_50_ to 40% of the value in ancestral cells, their sequential application would reduce IC_50_ to 16%. We note that such null models are imperfect, as they fail to capture epistasis and known hysteresis in evolutionary trajectories (Barbosa et al. 2017). Here, we use the null model only to identify candidate condition pairs for further experimental investigation. Under these additivity assumptions, the number of sensitivities is expected to increase for most pairs of stressors; that is, assuming additivity of the measured sensitivity profiles, sequential exposure to pairs of stressors is often predicted to sensitize the population to more stressors than exposure to either single agent alone (fig. 4*A*). In three cases (LZD–NaCl, LZD–NaBz, and NIT–SPT), the number of sensitivities is expected to increase by 3 or more, providing a substantial benefit over the single-agent selecting conditions.

To test these predictions experimentally, we focused on the pair LZD, a protein synthesis inhibitor, and NaBz, a commonly used food preservative. Our original selection experiments showed that selection in LZD led to five sensitivities and NaBz led to four sensitivities; the sensitivities are largely nonoverlapping, and sequential selection is therefore predicted to an increase in the number of sensitivities. To test this prediction, we performed experimental evolution on eight replicate populations to each of three conditions: LZD alone, NaBz alone, and a two-phase sequence consisting of LZD evolution followed by NaBz evolution. For convenience, we limited each evolution phase to 10 days (70–80 generations), making this considerably shorter than the original adaption in figure 1. We then tested an isolate from each population for modulated resistance to each of the 13 environmental conditions (fig. 4*B*).

The isolates selected by LZD or NaBz alone had sensitivity profiles that are similar, but not identical, to those selected in the original experiment (fig. 1). For both conditions, the single-agent evolution led to increased sensitivity to an average of approximately four conditions (fig. 4*C*). Strikingly, however, evolution in the LZD–NaBz sequence (“switch”) sensitized the isolates to more than six conditions on average, with some isolates exhibiting sensitivity to eight conditions.

To test the quantitative accuracy of the null model, we generated an ensemble of plausible resistance profiles for the sequential selection experiment. Each profile in the predicted ensemble corresponds to the mean of one pair of profiles, with one member of the pair drawn from the LZD-only selection (fig. 4*B*, left) and one drawn from the NaBz-only selection (fig. 4*B*, middle). The mean profile in this ensemble agrees surprisingly well with the mean profile measured in the LZD–NaBz evolution (fig. 4*D*).

## Discussion

These results provide a system-level picture of the phenotypic trade-offs accompanying evolved resistance to antibiotic and nonantibiotic stressors in an opportunistic pathogen. We find that collateral resistance and collateral sensitivity are surprisingly pervasive across conditions, underscoring the need to better understand how adaptation to nonantibiotic environments may contribute to drug resistance. These widespread collateral effects raise the question of whether frequently encountered stressors—food additives, preservatives, biocides, or simply common elements of natural environments—may steer bacteria toward multidrug resistance, and in turn, whether there may be an unappreciated role for these agents in slowing or reversing resistance. As proof-of-principle, we showed experimentally that sequential adaptation to different environments can be used to sensitize bacteria to antibiotics, a consequence of the largely nonoverlapping sensitivities induced by each agent alone.

The goal of this study was to investigate patterns of resistance between antibiotics and nondrug stressors at a phenotypic level. By taking a system-level view, we hoped to gauge the prevalence of collateral sensitivity and assess the potential of nonantibiotic agents for modulating resistance. This approach comes with obvious drawbacks, and it indeed leaves us with many unanswered questions. Most notably, it is vitally important to understand the molecular and genetic mechanisms facilitating these overlapping resistance profiles, though doing so on a broad scale is not easily done in one study. There are many well-known examples of molecular mechanisms that confer nonspecific collateral resistance to structurally unrelated compounds in bacteria, including a number of multidrug-resistant transporters and efflux pumps (Levy 1992; Lee et al. 2003; Kumar et al. 2013; Schindler and Kaatz 2016). Collateral sensitivity, on the other hand, remains much less understood, even simply between antibiotics. Recent evidence suggests that these sensitivities may be governed by target mutations that induce global changes in gene regulation or by mutations altering drug uptake and efflux (Pál et al. 2015). Similar mutations appear in our evolved mutations, suggesting that these mechanisms may also underlie many of the observed collateral effects between antibiotic and nonantibiotic stressors. However, definitely linking particular mutations with phenotypic effects will require considerable follow-up work to disentangle, for example, the potential effects of mutational epistasis and genetic background on drug-resistant phenotypes. In addition, although we used standard alignment protocols, identifying repeat sequences or mobile element mutations such as insertion sequences remains a challenge. Because mobile elements play an important role in V583 evolution, it is likely we were unable to identify some important mutations.

Additionally, it is not clear how these results might change if experiments were performed in a different ancestral strain. Most notably, strain V583 is highly resistant to multiple antibiotics; adaptation dynamics in strains without high-level multidrug resistance could differ substantially. Indeed, recent work underscores the notion that the ability to evolve antibiotic resistance depends on genotype (and therefore potentially history) (Card et al. 2019). Finally, we note that because we have limited the sequencing analysis to mutations that appear relatively frequently (>30%), our analysis omits some features of population heterogeneity that may play an important role in the evolution of collateral sensitivity. Future work may aim to further investigate links between this heterogeneity and the potential for gene-specific variations in mutation rates and selection.

We have shown experimentally that sequential adaptation to antibiotic and nonantibiotic conditions can sensitize bacteria to more environments than either agent alone. Although we focus here on a clinically relevant bacterial species, it is not clear that these results will generalize to other species. We used a simple additive model to identify candidate environmental pairs for sequential selection. Although the model gave surprisingly accurate predictions in these experiments, it will clearly fail when effects of epistasis or evolutionary hysteresis are strong (Roemhild et al. 2018). On the other hand, if epistasis effects are approximately symmetric about zero or typically small, additive null models—similar, in spirit, to those developed for drug combination effects (Russ and Kishony 2018)—may still prove useful for finding environmental pairs that increase the number of sensitivities, though the predictions of specific profiles are likely to become increasingly inaccurate. Long-term application will therefore require continued experimental mapping of the collateral sensitivity profiles selected by increasingly complex and realistic environmental conditions.

## Materials and Methods

### Strains, Antibiotics, Nonantibiotics, and Media

All mutants were derived from *E. faecalis* V583, a fully sequenced vancomycin-resistant clinical isolate (Sahm et al. 1989). The 13 conditions used to select mutants are listed in table 1. Antibiotics were prepared from powder stock and stored at −20 °C with the exception of AMP, which was stored at −80 °C. TCS, CHX, and NaBz were prepared from powder stock and stored at −20 °C. Acid (pH≈1.5) and base (pH > 10.5) stock solutions were prepared by titrating HCl and NaOH, respectively, into BHI medium. These stock solutions were mixed in appropriate volumes with standard BHI (pH≈7) to create selecting media for evolution experiments. Saturated KCl and NaCl stock solutions were prepared by dissolving KCl and NaCl into BHI medium. As with acid and base, appropriate mixtures of saturated KCl and NaCl solutions were mixed with standard BHI medium. Evolution and IC_50_ measurements were conducted in BHI medium alone.

### Laboratory Evolution Experiments

Evolution experiments were performed in 96-well plates with a maximum volume of 2 ml and a working volume of 1 ml BHI. Each day, at least three replicate populations were each grown in a different concentrations of the selecting agent. The concentrations were chosen to include both sub- and superinhibitory concentrations. After 20–23 h of incubation at 37 °C, aliquots (5 *μ*l) from the population that survived (OD > 0.3) the highest concentration were added to a new series of wells and the procedure was repeated for 30–60 days (maximum of about 450 generations). Note that isolates from antibiotic selection experiments were evolved for only 8 days (maximum of 60 generations), because resistance to antibiotics increased much more rapidly than resistance to other agents (Maltas and Wood 2019). To achieve roughly similar levels of resistance to the nonantibiotic conditions, we extended the selection time window until either 1) we observed 2–3 days of growth in a concentration at least two times the ancestral minimum inhibitory concentration or 2) the resistance appeared to plateau. Adaptation experiments to CHX and TRC lasted for 30 and 50 days, respectively, whereas NaCl, KCl, acid, base, and NaBz experiments continued for a total of 60 days. On the final day of selection, we plated a sample from each population on BHI agar plates, isolated a single colony from each plate, and stored the remaining population volume at −80 °C in 30% glycerol.

### Measuring Drug Resistance and Sensitivity

IC_50_ measurements for each condition/drug were performed in triplicate for each isolate (except in the case of the ancestral wild-type strain, which was performed in replicates of 8) in 96-well plates by exposing mutants in different wells to 6–10 concentrations of drug, typically in a linear dilution series prepared in BHI medium. After 12 h of growth at 37 °C, the optical density at 600 nm (OD) was measured using an Enspire Multimodal Plate Reader (Perkin Elmer) with an automated 20-plate stacker assembly.

Each OD reading was normalized to the OD reading for the same isolate in the absence of drug. To quantify resistance, the resulting dose–response curve was fit to a Hill-like function $f(x)={\left(1+{\left(x/K\right)}^{h}\right)}^{-1}$ using nonlinear least squares fitting, where *K* is the half maximal inhibitory concentration (IC_50_) and *h* is a Hill coefficient describing the steepness of the dose–response relationship. A mutant strain was deemed collaterally sensitive (resistant) if its IC_50_ varied by more than $3\sigma_{\text{WT}}$ from that of the ancestral strain, where *σ*_WT_ is the uncertainty (standard error across eight replicates) of the IC_50_ measured in the ancestral strain. Note that all estimates of IC_50_ in the ancestral (“wild-type”) strain, across all replicates and for all conditions, are contained in this $\pm 3\sigma_{\text{WT}}$ range, which suggests that there are unlikely to be false positives in designating isolates as sensitive or resistant.

### Whole-Genome Sequencing

We sequenced single isolates and population samples from the 13 evolved populations and a control V583 strain propagated in BHI for 8 days. We also sequenced single isolates from two (ancestral) V583 frozen stocks. Samples from each population were streaked from a frozen stock, grown overnight in BHI, and triple washed in phosphate buffered saline. DNA was isolated using a Quick-DNA Fungal/Bacterial Kit (Zymo Reserach) according to manufacturer’s instructions. The clonal samples were sequenced at the University of Michigan sequencing core using an Illumina MiSeq system, and the population samples were sequenced at the Microbial Genome Sequencing Center (MiGS) at University of Pittsburgh using a NextSeq 550 system.

The resulting genomic data were analyzed using the high-throughput computational pipeline breseq (Deatherage and Barrick 2014), with default settings. Average read coverage depth was about 150 for single-colony sequencing and 200 on the population sequencing batch. Briefly, genomes were trimmed and subsequently aligned to *E. faecalis* strain V583 (accession numbers: AE016830–AE016833; see Paulsen et al. 2003) via Bowtie 2 (Langmead and Salzberg 2012). A sequence read was discarded if <90% of the length of the read did not match the reference genome or a predicted candidate junction. At each position, a Bayesian posterior probability is calculated and the log10 ratio of that probability versus the probability of another base (A, T, C, G, gap) is calculated. Sufficiently high consensus scores are marked as read alignment evidence (in our case, a consensus score of 10). Any mutation that occurred in either of the two control V583 strains was filtered from the results.

For population sequencing, we limit our analysis to mutations that occur at a frequency of >30%. The choice of a cutoff percentage will always be slightly arbitrary. Our goal was to minimize false positives while maintaining the ability to identify mutations that occur at <100%. Our choice of 30% stems from the distribution of mutation frequencies from our population sequencing sample (see supplementary fig. S3, Supplementary Material online); below 30%, the number of variant calls rises rapidly. Although it is possible, and perhaps likely, that lower-frequency mutations may play a significant role in the evolution and observed phenotypes, additional experiments would be needed to distinguish true variants from false positives.

## Supplementary Material

Supplementary data are available at *Molecular Biology and Evolution* online.

## Supplementary Material

msz303_Supplementary_Data

## Notes

All sequencing data were deposited at NCBI’s short read archive and are accessible with BioSample accession number SAMN13383020, project ID: PRJNA591549.
