# Supplementary material for: Using Selection by Nonantibiotic Stressors to Sensitize Bacteria to Antibiotics
Source: Mol Biol Evol. 2019 Dec 18;37(5):1394–406. doi: 10.1093/molbev/msz303 (PMC7182213; doi:10.1093/molbev/msz303)
Supplement: msz303_Supplementary_Data [file msz303_supplementary_data.zip › msz303-Suppl_Data/SupplementalFiguresEnvMaltas.pdf]

Supplemental Material

The Supplemental Material contains 4 supplemental figures (S1-S4).

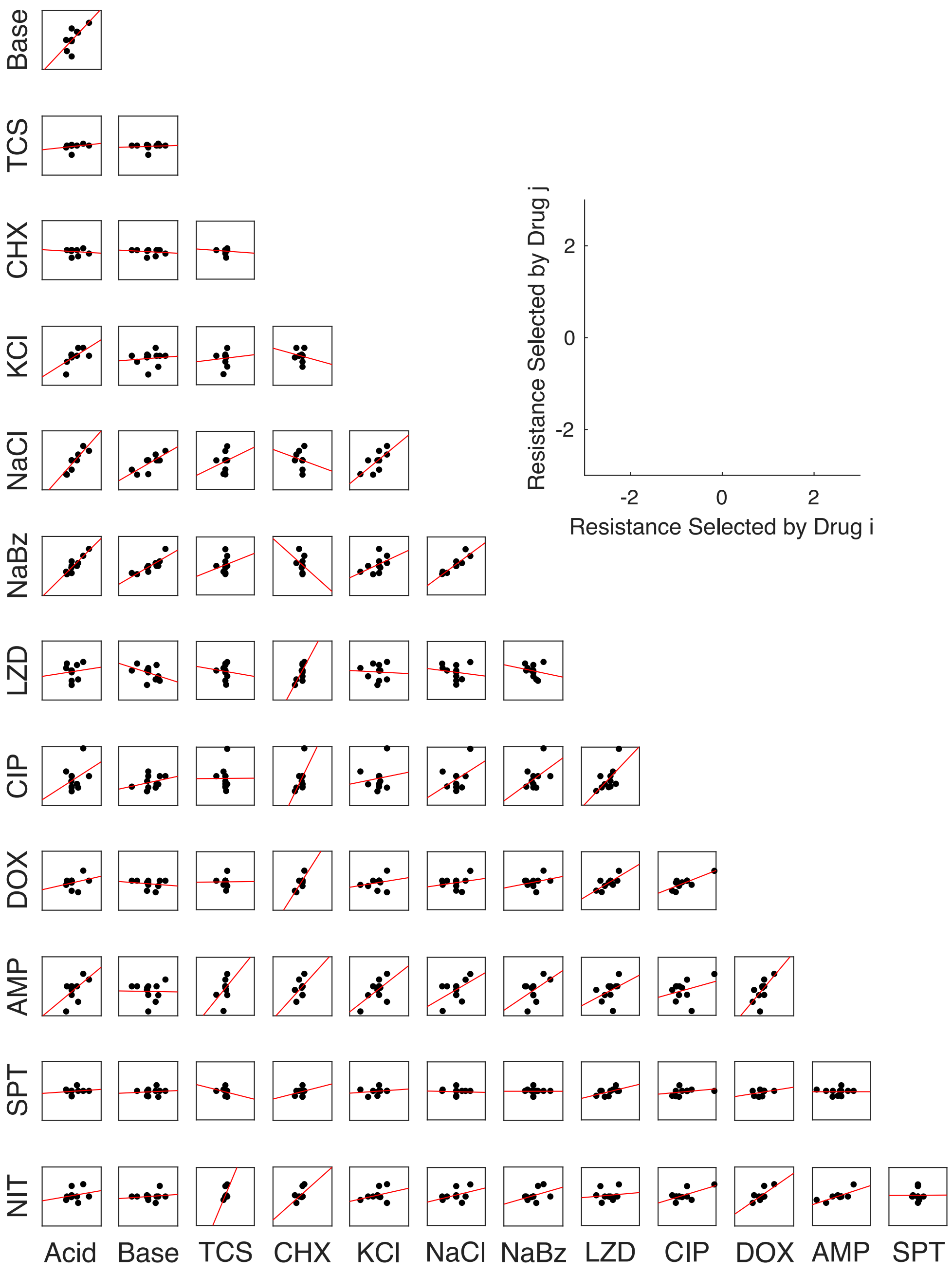

FIG. S1. Scatter plots for resistance levels selected by different pairs of conditions. Pairwise scatter plots of resistance profiles selected by different conditions (that is, scatter plots comparing pairs of columns of the collateral sensitivity matrix). Each point represents the measured resistance to a single stressor in isolates selected by the pairs of conditions on the horizontal and vertical axes. To remove the effects of direct selection, which are typically larger in magnitude and may bias the correlations, the diagonal entries of the collateral sensitivity matrix (corresponding to resistance to the selecting condition) are removed. In all cases, resistance is measured in units of (log_2-scaled) fold change in IC_{50} relative to ancestral strain.

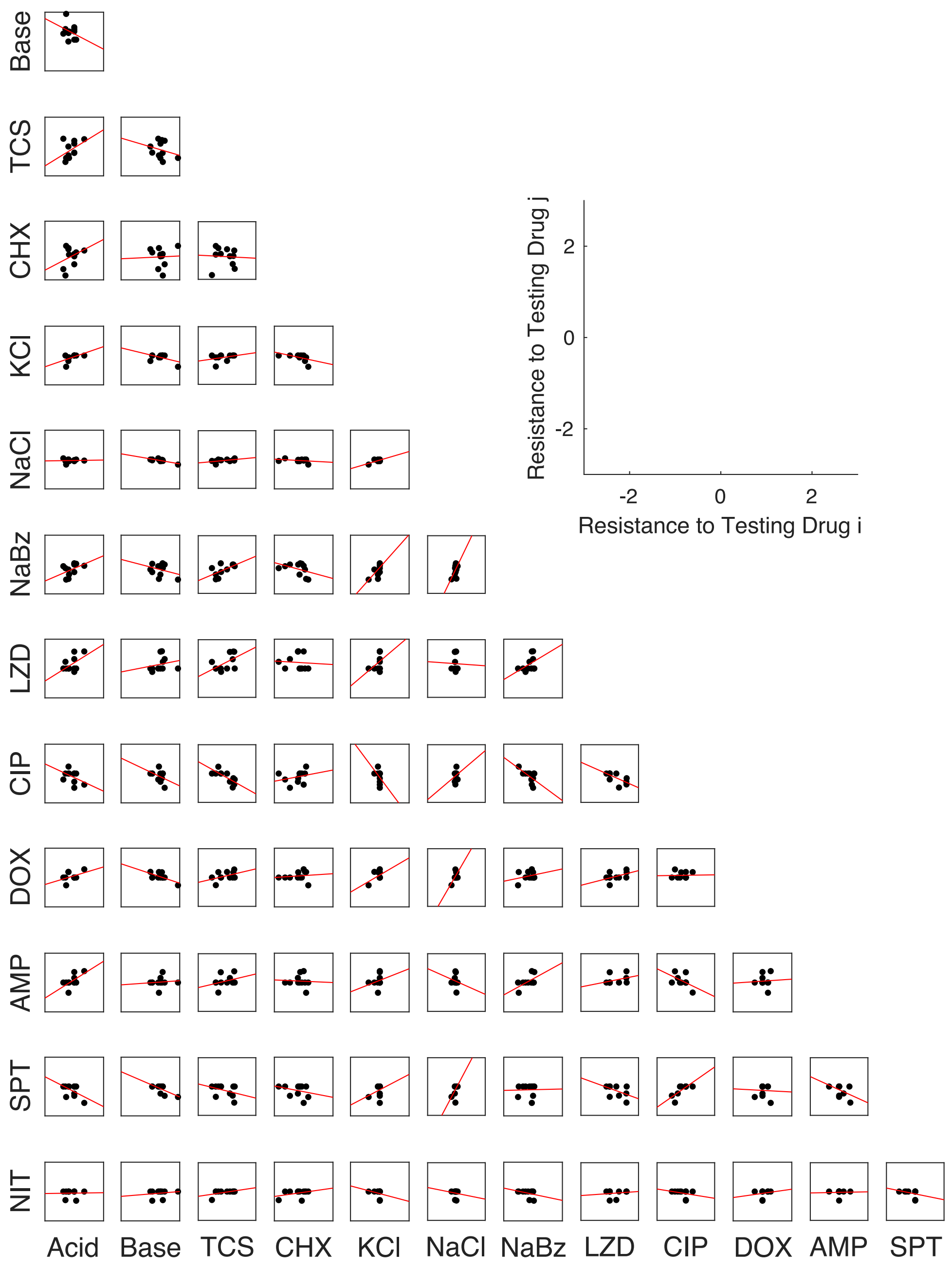

FIG. S2. Scatter plots for resistance levels to different pairs of testing conditions. Pairwise scatter plots of resistance levels to different conditions (that is, scatter plots comparing pairs of rows of the collateral sensitivity matrix). Each point represents resistance to each of the paired testing conditions (on the horizontal and vertical axes) in a single isolate. To remove the effects of direct selection, which are typically larger in magnitude and may bias the correlations, the diagonal entries of the collateral sensitivity matrix (corresponding to resistance to the selecting condition) are removed. In all cases, resistance is measured in units of ($\log_2$-scaled) fold change in IC_{50} relative to ancestral strain.

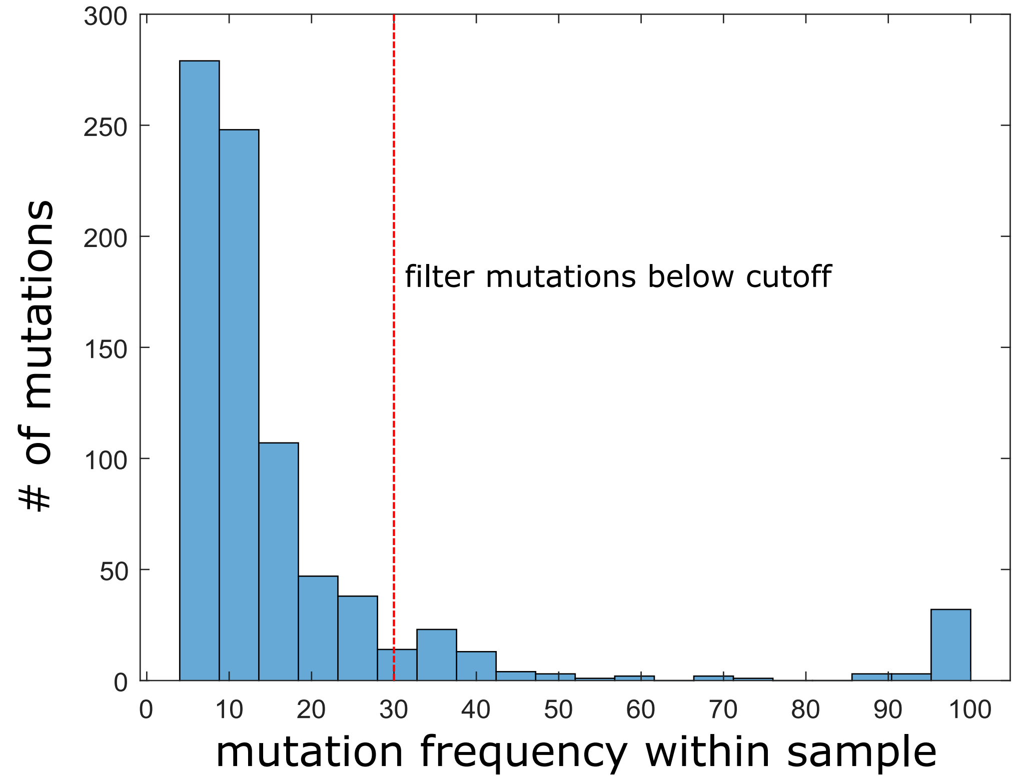

FIG. S3. Distribution of frequencies from identified mutations via population sequencing. A histogram revealing the sample frequency of each mutation identified via population sequencing. The dotted red lines denotes the semi-arbitrary cutoff we have chosen where any mutation with a frequency below 30 percent is filtered from analysis.

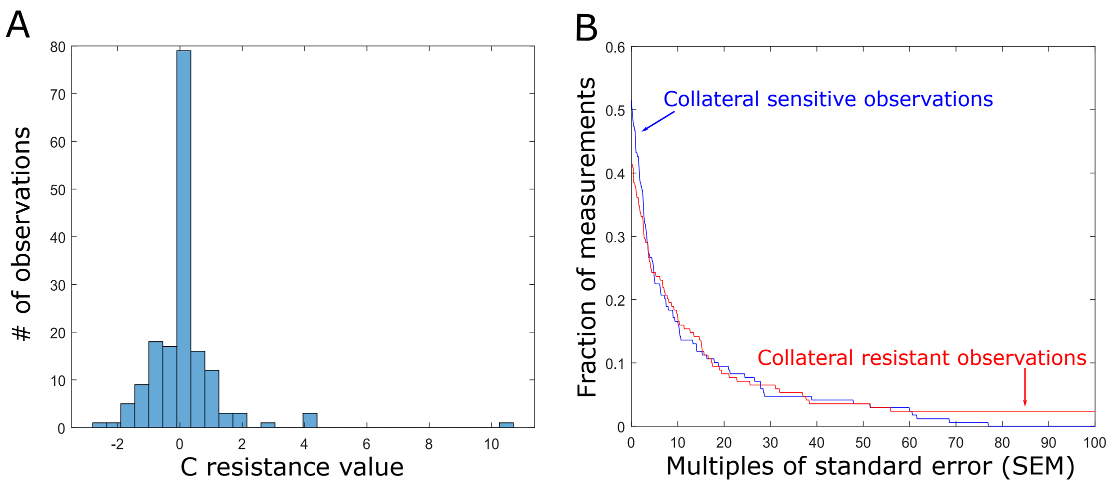

FIG. S4. Distribution of resistance values, c, across all mutants. A. Histogram revealing the frequency of measured resistance values across all 13 mutants. B. Ratio of the number of CS observations to the number of CR observations as a function of c-value cutoff, where $c \equiv \log_2\left(\mathrm{IC}_{50,Mut}/\mathrm{IC}_{50,WT}\right)$
