# Supplementary material for: Using Selection by Nonantibiotic Stressors to Sensitize Bacteria to Antibiotics
Source: Mol Biol Evol. 2019 Dec 18;37(5):1394–406. doi: 10.1093/molbev/msz303 (PMC7182213; doi:10.1093/molbev/msz303)
Supplement: msz303_Supplementary_Data [file msz303_supplementary_data.zip › msz303-Suppl_Data/Table_MutationsEnviopaperSized.pdf]

Sample	Gene	Pop (%)	Clonal	Description
AMP*	*EF_3191* ← / → *EF_3192*	100	✓	Intergenic
	pyrR ←	43.1		Regulates pyrimidine biosynthesis
	hexA ←	31.7		DNA repair protein
	EF_3290 →	93	✓	Sensor histidine kinase
BASE	*EF_0096* ← / → *EF_0097*	100	✓	Intergenic
	recU ← / → *EF_1150*	100	✓	Intergenic
	EF_1711 ← / ← *pyrE*	100	✓	Intergenic
	ccpA ← / → *pepQ-2*	100	✓	Intergenic
	EF_1204 → / ← *EF_1206*	58.4		Intergenic
	EF_1936 ←	60.6		Conserved hypothetical protein
	EF_3014 ← / ← *EF_3015*	94	✓	Intergenic
CIP	***parC*** ←	100	✓	Topoisomerase IV
	EF_0184 → / → *deoB*	31.9		Intergenic
	[EF_tRNALeu3]–[EF_1076]	100	✓	24-gene deletion
DOX*	*rpsJ* →	100	✓	30S ribosomal protein S10
	rpsJ →	87	✓	30S ribosomal protein S10
	prgB →	100	✓	Surface aggregation protein
CHX	*EF_1608* ←	100	✓	Cardiolipin synthetase
	EF_2227 →	100	✓	ABC transporter
	rpoC ←	100	✓	RNA polymerase
	EF_1187 →	30.6		Conserved hypothetical protein
	EF_1456 →	68.3	✓	Conserved hypothetical protein
	EF_1570 →	38.9		Conserved hypothetical protein
	EF_3114 ←	74.7	✓	Conserved hypothetical protein
KCl	EF_1096 → / → EF_1097	100	✓	Intergenic
	**vicK** →	100	✓	Sensor histidine kinase
	**galU** ←	100	✓	UDP-glucose pyrophosphorylase
	EF_1789 ←	100	✓	Conserved hypothetical protein
	EF_2348 ←	100	✓	Conserved hypothetical protein
	EF_2886 ←	100	✓	*marR* family transcriptional regulator
	recA ←	100	✓	DNA repair protein
	EF_0871 → / → *EF_0872*	40.6		Intergenic
	glnA ←	36		Glutamine synthetase
LZD	*EF_1414* ←	100	✓	Conserved hypothetical protein
	EF_0149 → / ← *EF_0150*	34.1		Intergenic
	EF_0797 ←	66.6		Conserved hypothetical protein
	EF_0871 → / → *EF_0872*	33.8		Intergenic
	EF_0871 → / → *EF_0872*	49.4		Intergenic
	prgB →	78.8		Surface aggregation protein
NaBz	***EF_1148*** ←	100	✓	Penicillin binding protein 1A
	atpD ←	100	✓	ATP synthase F1, beta subunit
	EF_0871 → / → *EF_0872*	32.6		Intergenic
	EF_0871 → / → *EF_0872*	31.2		Intergenic
	EF_2604 ←	89.6		Conserved hypothetical protein
NaCl	***vicK*** →	100	✓	Sensor histidine kinase
	codY ←	100	✓	Transcriptional regulator
	EF_2886 ←	100	✓	*marR* family transcriptional regulator
	galU ←	91.8	✓	UDP-glucose pyrophosphorylase
SPT*	*rpsE* →	100	✓	40S ribosomal protein S5
TCS	***EF_0142*** →	100	✓	Multi-drug efflux pump
	fabI ←	100	✓	Triclosan target
	EF_1972 ←	100	✓	Conserved hypothetical protein
	EF_1151 → / → *EF_1152*	37.9		Intergenic
	EF_3074 ←	34		Conserved hypothetical protein
